# Visual Health Analysis of Print Advertising Graphic Design Based on Image Segmentation and Few-Shot Learning

**DOI:** 10.1155/2022/8040913

**Published:** 2022-03-24

**Authors:** Sen Hu

**Affiliations:** School of Design,Shandong University of Arts, Jinan,250307, China

## Abstract

Graphics innovation must adapt to the changing trends of the times in order to stay ahead of the curve. This article investigates the use of graphic vision in print advertising design, using image segmentation as a starting point and examines the current state of visual innovation in print advertising graphic design and its various expressions and applications. A series of homogeneous regions are generated and outlined using an image segmentation algorithm based on adaptive local threshold. The user then paints different colors on the area sets that make up the various targets. Finally, the image segmentation is completed by merging the color mark region sets. Automatic extraction of the initial curve of an active contour model, construction of an active contour model based on saliency and level set solution, automatic selection of training samples when a classifier is used for image segmentation, and so on are all problems that this method effectively solves. Experiments show that this algorithm not only satisfies users' demands for more intuitive input and more accurate interactive image segmentation results but also enables multiregion and multitarget image segmentation with ease.

## 1. Introduction

Graphics and image processing [1] has become one of the most active research directions today as a result of the advancement and perfection of computer science and related mathematical theories. Among them, graphic image segmentation has long been a hot topic in this field. Image segmentation is the process of dividing an image into several disjoint regions based on a similarity measurement criterion, with pixels in the same region having similar features and pixels from different regions having low similarity [2]. Graphic image segmentation technology has found widespread use in a variety of fields, including computer animation, medical image processing [3], virtual reality, computational visualization, and so on. Graphics have become synonymous with fashion in the irresistible visual space. Changing time and space gives graphic vision a new lease on life, as well as revealing its glory. Many image processing tasks necessitate the extraction of some image areas. We can divide the pixels by means of image segmentation [4, 5] technology and separate the target areas from the background. At present, many image segmentation methods have been proposed, but there are still some problems, such as poor universality, dependence on user interaction, and inconsistency between segmentation results and human visual perception. With the deepening of the era of reading pictures and the development of artistic interoperability, the aesthetic ability and demand of the public are further improved, and the print advertising design industry is facing new challenges. Graphics in modern print advertisements are intuitive, vivid, lively, distinctive, unique, and changeable. The language displayed by graphics can be our perception of daily life or the trend of the times [6].

Print advertising is an important part of today's social and economic life, and it helps to promote the circulation of goods [7]. Every designer working in print advertisement design is concerned about how to make print advertisements better serve today's society [8]. Currently, issues such as design material simplification and homogenization are more prominent in the industry. Design practitioners are concerned about how to use image processing technology to improve the innovation of print advertising design [9]. The data item established by the local algorithm is the first part of the energy function of the traditional graph cutting algorithm, and the smoothing term of disparity difference between neighboring pixels is the second part. The local algorithm data items will cause relatively large errors due to occlusion and other reasons, and the smoothness assumption of occluded areas is often not valid, so parallax mismatch will occur in some occluded areas [10]. With people's access to images becoming more convenient and quick, more image segmentation algorithms are being used for Internet and mobile device image processing tasks like image classification, marking, retrieval, and thumbnail generation, among others, which necessitates image segmentation algorithms that are fast, accurate, and have the advantages of automatic and real-time processing [11]. Image segmentation is a key step from bottom image processing to image recognition and understanding because the result has a direct impact on subsequent visual tasks [12]. Humans can accurately separate the target area in an image, but computers find it difficult. This article investigates and analyzes the visual innovation of graphic design in print advertisements using image segmentation technology.

At present, in this era of visual transmission everywhere, our sights or thoughts are usually filled with various kinds of graphics [13]. Graphics spread information everywhere in the world, and it has become an expression language without national boundaries. On the one hand, modern print advertising is a new way to promote commercial advertising; on the other hand, it is also the expression of aesthetic symbolism in the market economy [14]. Graphic vision in modern print advertising design, as a combination of technology and art, embodies the diversity and richness of modern culture all the time. As a force that cannot be ignored in visual art, it promotes the development of modern print advertising design and promotes print advertising design to enter a new visual era. This kind of commercial works combined with artistic sense can often stimulate the infinite imagination of the audience, and at the same time, it will leave a deep impression on the audience [15]. This will also greatly promote the products involved in the advertisement. Graphic vision in graphic advertising design is not just a picture to convey information, but a kind of symbolic sentiment, which can deeply attract people, move people, and inspire people. Graphic vision will show different advertising themes according to different advertising concepts [16]. As print commercial advertising is an outstanding performance of innovation, the graphic design of print advertising not only plays the role of information transmission in external publicity but also is a prominent performance of corporate culture. Based on image segmentation, this study analyzes the innovation of graphic design vision in print advertisements and discusses how graphic design vision will face the public. The potential position of the target object is predicted using a saliency map, and the result is further refined using a morphological operation to obtain the target object's contour information. The objective function is defined to quantitatively describe the visual information in an image based on human visual characteristics. The global optimal threshold of each subgraph is obtained by optimizing the objective function. The final threshold segmentation result is then obtained by performing a local adaptive threshold operation using the image's local characteristics. The results of the experiments show that this method is active and adaptive. Furthermore, the segmented binary image has a pleasing visual effect, and its overall visual quality is closer to that of human perception.

## 2. Related Work

People are tired of traditional marked graphics, according to Zhang et al. [17], and the era of traditional print advertising is over. According to Wang [18], it is necessary to combine innovative ideas in advertising art with technical science, as well as to demonstrate cultural diversity, while considering the economic benefits of advertising. In order to encourage in-depth visual concept innovation in print advertising design, discussing the importance of graphic vision and studying the innovative elements of graphic vision in print advertising design, according to Walter et al. [19], will help graphic vision better serve people and encourage them to use graphic visual language more quickly. Soldow [20] discussed iconology and conducted a pedigree study on the visual images used in print advertisements. The visual images used in print advertisements are systematically classified and studied in groups as a result of this. According to Ramezani et al. [21], graphic image processing is usually based on graphic image segmentation technology, which allows the target area to be accurately separated from the graphic image background. Javan and Zeman [22] proposed a new interactive image segmentation algorithm. This algorithm overcomes the shortcomings of many current interactive algorithms, such as cumbersome operation and ineffective multiobjective segmentation and is very practical. Based on the analysis and research of existing graphics and image segmentation algorithms, combined the characteristics of human vision and related theories with the segmentation process, Miller et al. [23]proposed new graphics and image segmentation algorithms, respectively. Zeller [24] compared the visual images of graphics in print advertising design in different times. At the same time, through the analysis of the development process of print advertising, the connotation of graphic vision in modern print advertising design is interpreted.

This article summarizes the composition and characteristics of the human visual system and comprehensively analyzes the problems that exist in current graphic image segmentation based on an in-depth review of related literature. A new adaptive image segmentation method is proposed that combines the threshold selection process with human visual characteristics. The geometric attributes of the mesh model, which include arc length, angular distance, and correction term, define the distance function between mesh vertices. Visually meaningful segmentation results are obtained by clustering the grid's vertices. This research combines print advertising design with image processing technology, resulting in a unique artistic effect and increased advertising information transmission accuracy. It is also more in line with the current aesthetic trend, and it is more conducive to the spread of digital network media and other forms of communication.

## 3. Methodology

### 3.1. Visual Innovation in Graphic Design of Modern Print Advertisements

Vision is one of the important ways for human beings to obtain information about the surrounding environment. The human visual system generally consists of two parts: the human eye and the central nervous system of vision. Among them, the human eye is responsible for perceiving visible light information, which projects a two-dimensional image on the human retina [25]. The visual central nervous system is responsible for the reconstruction, positioning, discrimination, analysis, and research of two-dimensional images. Visual perception is a highly complex visual perception process, which is from the direct response to the attributes of the target object to the acquisition of its advanced semantic information. At present, many methods in graphics and image processing are based on mathematics, computer, and other disciplines. Because human intuition and analysis of things will play an important role in the process of selecting methods, this selection process is usually based on human subjective visual judgment. Therefore, understanding the composition of human visual system and its visual characteristics is of practical significance for solving the problems in graphic image processing.

**Figure 1:**
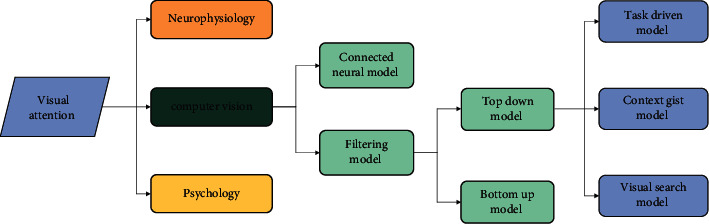
The category of attention mechanism.

In modern society, due to the progress of science and technology, all kinds of images that human beings can see have far exceeded the sum of the visual images of any era after the emergence of human beings. The visual attention mechanism [26] helps the brain to process all kinds of information in the visual field in parallel, and visual salience can help the visual attention system to realize automatic real-time target selection. After calculating the saliency of each object in vision, the vision system will use different eye movement strategies to determine the attention order of several prominent objects in vision, and then transfer attention among these prominent objects [27]. The main feature of human visual concentration is that the human visual system can quickly fix the visual attention on several obvious visual targets that it is interested in and automatically screen out other visual targets when faced with a large number of complicated visual information. When dealing with complex scenes or facing a large amount of visual information, the human visual system can quickly focus on several prominent targets while ignoring other uninterested objects. This process is called visual attention mechanism. The visual mechanism is shown in Figure 1.

In modern print advertising design, the creative elements of graphic vision are important in design expression techniques. Each designer may have different ways and means of conceiving and express the connotation of print advertising works through graphic vision creativity. To study the visual innovation of graphic design, we must analyze it from the most fundamental elements. This kind of element is the tool of creation. Visual creativity refers to the expression of creative ideas [28]. In the graphic design of contemporary print advertisements, the visual innovation elements of graphic design play an important role in the way of expression. Different designers have different ways of thinking and show the specific meaning of advertising works by means of graphic design visual creativity. In the design of modern print advertisements, graphic creative elements commonly used can be divided into abstract graphic creative elements and realistic graphic creative elements.

In a broad sense, visual image refers to all images that human beings see. The premise is to have a normal visual system. Images in nature are complicated and rich. As a specialized visual discipline, the visual image of print advertisement needs more strict classification standards. Classifying all kinds of images will help us to better grasp the fundamentals of image application. Among the graphic design elements, the design of Chinese and English fonts is quite common [29]. Chinese characters are the accumulation of Chinese traditional culture, and characters are one of the best graphic elements to accurately convey information. Usually, designers design graphic visual creativity from the pictographic consciousness of characters. The creativity of Chinese characters is to re-create graphics on this basis. Using the product of modern social development, modern element symbols, and regraphics and creativity in the structure of Chinese characters, people can imagine another meaning after the change in the basis of familiarity with existing Chinese characters.

**Figure 2:**
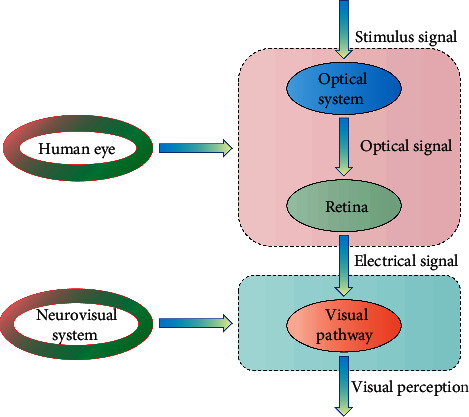
Schematic diagram of visual information processing model.

Graphic vision is an important way of expressing oneself in the graphic creation of contemporary print advertisements, and it is the focus of the audience's attention. Good advertising creation can reveal the ideas to be expressed in advertising creation using its own distinct graphic language and can succinctly and effectively show the theme with profound meaning [30]. Graphic advertisement, as a concrete manifestation of graphic design, bears most of the design features of graphic design, particularly the design innovation of graphics and pictures, which plays a significant role in the design process. Graphic design vision, in particular, is the soul of print advertising design. To fully comprehend the overall design work, start with these subtle elements, consider graphic elements as the building blocks of graphic design, and consider the visual effect of the results as the standard for testing the properties of graphic elements. The premise foundation of this type of advertising product is innovative ideas of vision and creative forms of expression of ideas. With the increasing variety of visual expression creation, graphic design's visual language should use new ways to realize ideas in the creation process. It is clear that graphic vision creation plays a vital role in the graphic creation of contemporary print advertisements. The graphic creation elements in the graphic creation of print advertisements are also investigated, and it can be concluded that graphic vision creation plays a vital role in the graphic creation of print advertisements. In the current graphic design process, the graphic elements involved are abstract and concrete. Analyzing and grasping these two elements will promote the development of the whole print advertisement. The visual information processing model is shown in Figure 2.

Visual traction is a bright spot in the graphic design process of current print advertisements. There are two aspects to draw the visual audience's information: using color and using graphics, the audience can follow the designer's ideas to appreciate the works. Most existing print advertisements are guided by dots, lines, and planes. The subjective initiative quota of people plays an important role in the design process. Different designers often design different graphics to express different artistic feelings as a result of their diverse life experiences, and even the same type of graphic design can be interpreted in different ways depending on the language used. Visual metaphorical expression in graphic works is an extremely important, unique, and inventive expression form in today's print advertising, and we can also use metaphorical expression techniques in graphic design of contemporary works in large quantities. This type of graphic expression can help to advance graphic vision in works, as well as focus the audience's attention, which can benefit businesses financially.

In the design of modern print advertisements, realistic graphics, a creative element, also occupies an important position, which is equally divided with abstract graphics. Although realistic graphics have not changed in various ways, it can make the audience feel the real sense of existence and narrow the sense of distance between audiences. Excellent image creation advertising works do not care how much the graphics change but the extraction and processing of creative thinking. No matter how the graphic design itself changes, we cannot interpret without the two scales of abstraction and concreteness. The correct use of these two tools will not only help the audience to understand the graphic design but also promote the inspiration of designers.

The idea of minimalism comes from the minimalism of contemporary school. The designers of this school all encourage minimalist drawing methods, and the purpose of the simplest treatment of creation is to find a simple scheme that is rich in content and pure in Chinese and modern classics. In order to create advertising works with more special visual effects, I should pay attention to the existence of minimalist style, master it, and use it properly in the visual creation of graphic works. Learn about graphic effects and how to use them. We should concentrate on displaying the bright spots of graphics during the graphic design and postdecoration processes in order to fully attract the audience's attention and increase their interest in graphic culture and persuade the audience to accept the advertisement's content while admiring and being drawn to the beauty.

### 3.2. Graphic Design and Implementation of Print Advertisement Based on Image Segmentation

Image segmentation has always been one of the most difficult problems in computer vision, with the ultimate goal of allowing computers to mimic human visual cognition and recognize the meanings represented by different regions in an image. Traditional image segmentation methods and image segmentation methods combined with new theories are the two main types of image segmentation methods. Early image segmentation methods relied on analyzing image features, such as grayscale, orientation, size, and shape. This category includes histogram threshold segmentation methods, region-based segmentation methods, edge-based segmentation methods, and hybrid segmentation methods. With the advancement of computer vision and research into human cognitive ability, the question of how to use human visual characteristics to produce more reasonable image segmentation results has become a hot topic in research.

The basic principle of threshold-based image segmentation method is as follows. Let the original image as *f*, the image size as *M* × *N*, the image grayscale as *L*, and *f* (*x*, *y*) represents the gray level of the pixel whose coordinates are (*x*, *y*),then *x* ∈ [1, *M*], *y* ∈ [1, *N*]. The single-threshold segmentation method is to determine a segmentation threshold *T*, segment the gray levels of all pixels, and obtain the segmentation result *g*(*x*, *y*):

(1) $$\begin{array}{c} g\left(x,y\right)=\left\{\begin{array}{cc} 0, & 0\leq f\left(x,y\right)\leq T,\\ L-1, & T<f\left(x,y\right)\leq L-1, \end{array}\right) \end{array}$$

where *T* is a constant that applies to the whole image, the processing method given by this formula is called global thresholding. When the value of *T* changes constantly and is determined by (*x*, *y*) and its neighborhood characteristics, the processing method is local thresholding. The multithreshold image segmentation method adopts multiple thresholds, set the number of thresholds as *n*, and denote *T*_1_, *T*_2_,…, *T*_*n*_ as the segmentation threshold, then the image *g*(*x*, *y*) after segmentation is

(2) $$\begin{array}{c} g\left(x,y\right)=\left\{\begin{array}{cc} L_{0} & 0\leq f\left(x,y\right)\leq T_{1},\\ L_{1} & T_{1}<f\left(x,y\right)\leq T_{2},\\ \cdots & \cdots \\ L_{n-1} & T_{n-1}<f\left(x,y\right)\leq T_{n},\\ L_{n} & T_{n}<f\left(x,y\right)\leq L-1, \end{array}\right) \end{array}$$

where *L*_0_, *L*_1_,…, *L*_*n*_ are the *n* + 1 gray levels of the segmented image.

There are four major flaws in the segmentation of two-dimensional images in the computer image processing process right now. (1) An insufficient number of image segmentation methods are available. (2) Image segmentation has a low level of accuracy. (3) There is no perfect quality evaluation standard for image segmentation. (4) Image segmentation results are difficult to match human visual perception requirements. The threshold segmentation method is a simple image segmentation method that uses the gray level difference between the target object and the background in the image to select one or more thresholds based on the image gray histogram and divides the image into target and background areas with different gray levels. The key to using a threshold segmentation algorithm is to pick the right one. When the gray histogram of an image has a bimodal shape, the gray value corresponding to the valley between two peaks is selected as the threshold, depending on the number of thresholds used in segmentation. Different thresholds or dynamic thresholds can be used in different positions of the image using variable and multiple thresholds. The visual mechanism is still under investigation due to the complexity of the human visual system. Many image segmentation methods currently use visual saliency as a visual mechanism, with the image's saliency map being used to simulate vision.

Interarea contrast GC indicates the gray contrast between the divided areas:

(3) $$\begin{array}{c} \text{GC}=\frac{\left(f_{1}-f_{2}\right)}{f_{1}+f_{2}}, \end{array}$$

where *f*_1_ and *f*_2_ are the average gray levels of pixels in the target area and the background area, respectively. Internal uniformity UM indicates the degree of uniform distribution of pixel characteristics in each area in the segmented image.

(4) $$\begin{array}{c} \text{UM}=1-\frac{1}{C}{\sum_{i=1}^{2}\sum_{\left(x,y\right)\in R_{i}}\left[I\left(x,y\right)-\frac{1}{A_{i}}\sum_{\left(x,y\right)\in R_{i}}I\left(x,y\right)\right]}^{2}. \end{array}$$

C is the normalization coefficient, and *R*_1_ and *R*_2_ represent the segmented target and background regions, respectively. *A*_1_ and *A*_2_ represent the areas of the target and background areas. Pixel distance error FOM is defined as follows:

(5) $$\begin{array}{c} \text{FOM}=\frac{1}{N}\sum_{i=1}^{N}\frac{1}{1+p\times d^{2}\left(i\right)}. \end{array}$$

Among them, *N* is the number of misclassified pixels, *p* is the scale coefficient, and *d*^2^(*i*) represents the distance between the *i*-th misclassified pixel and its correct position. Pixel segmentation error PE is the number of misclassified pixels due to segmentation errors:

(6) $$\begin{array}{c} \text{PE}=P\left(o\right)\times P\left(b|o\right)+P\left(b\right)\times P\left(o|b\right). \end{array}$$

Among them, *P*(*b|o*) and *P*(*b|o*) represent the probability of misclassifying the target pixel as the background, and the probability of misclassifying the background pixel as the target, respectively. *P*(*o*) and *P*(*b*) are the prior probabilities of the object and background in the image, respectively.

The original image is divided into multiple homogeneous connected regions by minimizing the energy function. Using the specific law of the boundary curve of the target object, the homogeneous region with small intensity changes is represented by a piecewise smooth function, although the intensity changes are drastic. The region's boundary is represented by the union of short smooth functions, with the discontinuous point set of the function close to the target object's boundary to achieve effective image segmentation. The algorithm's specific implementation process is divided into three steps: (1) divide the pixels in the image, (2) make a new two-dimensional image, and (3) determine the image segmentation threshold for the corresponding image. It is necessary to determine the corresponding objective function based on human visual characteristics and optimize the corresponding objective function in this link. Finally, it is combined with the image's local pixels to scientifically calculate an image segmentation threshold that is consistent with human visual characteristics, and the image is segmented.

The target area is extracted from the background area of the image by calculating and selecting the image threshold. Although the image separated by the threshold segmentation algorithm has a small amount of data, it occupies less storage space, which significantly simplifies the subsequent image analysis and image processing links, and speeds up the efficiency of image processing. Compared with the original image, the amount of binary image data generated by threshold segmentation is reduced, which can highlight the target area of interest, but the process of thresholding will also cause the loss of many other important information in the original image, thereby reducing the binary value and the visual quality of the image. Therefore, reducing the loss of main information in the threshold segmentation process is the key to improving the quality of the final binary image.

Based on the quality of the reconstructed image, peak signal-to-noise ratio measurement method is given as

(7) $$\begin{array}{c} \text{PSNR}=10\times \log_{10}\left(\frac{{\text{MAX}}_{i}^{2}}{\text{MSE}}\right),\\ \text{MSE}=\frac{1}{N}{\sum_{\left(x,y\right)}\left\|\text{Rco}\left(nx,y\right)-Or\left(ix,y\right)\right\|}^{2}, \end{array}$$

where MAX_*i*_ is the largest pixel in the image, Rcon is the reconstructed image, Ori(*x*, *y*) is the real image of the reconstructed image, and N is the total number of pixels.

The active contour model is founded on curve evolution theory and the level set method. The zero level set of the traditional level set function represents the contour curve. By updating the level set function, the model achieves its goal of moving the contour line. The level set function can remain effective even if the contour line undergoes topological changes such as splitting or merging. The algorithm uses human visual characteristics as a starting point for image segmentation, then uses advanced methods to optimize the image's segmentation threshold from a humanized perspective, and finally makes the segmented target 2D image better meet human visual perception needs. The algorithm automatically merges the target region sets according to these marks to complete the separation of the target from the background. Users can clearly and roughly mark the regions that make up the target.

## 4. Result Analysis and Discussion

Image segmentation gathers pixels in an image into different connected regions according to certain similarity criteria, and its purpose is to simplify the expression form of the image and extract more abstract and compact useful information from complex image information, which is beneficial to subsequent processing and analysis. The key of threshold segmentation method is how to select the appropriate threshold, which will directly affect the final segmentation result. We use the optimization method to solve the optimal threshold of the image, so as to give visual meaning to the threshold and then combine the local properties of the image to produce threshold segmentation results that are more in line with human visual perception.

**Figure 3:**
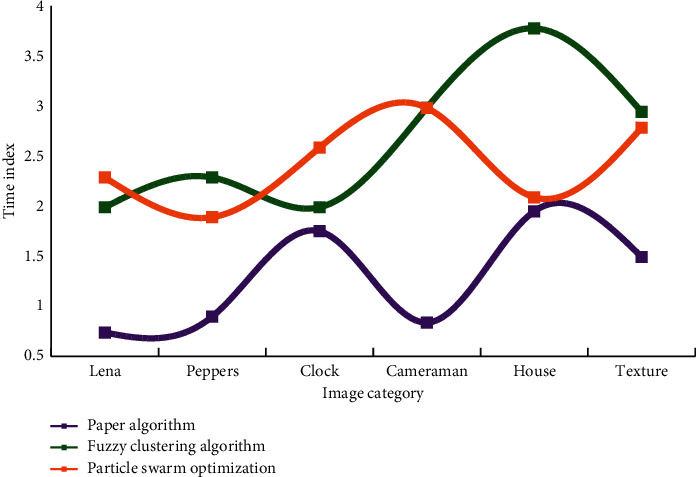
Comparison of calculation time of three algorithms.

When people observe a scene, the processing unit is not pixels, but an area or outline composed of many pixels, according to the working principle of the human visual system. Pixels are merely a representation of the transition from continuous to discrete in the image digitization process, and they do not exist in the human visual system or brain. People can only have visual perception significance when pixels are combined. Experiments are used to verify the effectiveness of the adaptive threshold segmentation method proposed in this chapter, and we have verified a large number of images and did subjective visual comparison of the experimental results, as well as quantitative comparison using various image quality evaluation indicators. The process of calculating the global optimal threshold for each subgraph is the most time-consuming part of the algorithm. Before we can calculate the optimal threshold for each subgraph, we must optimize the objective function twice. The time comparison of the algorithm proposed in this article with fuzzy clustering and particle swarm optimization algorithms is shown in Figure 3.

Experiments show that compared with the calculation time of particle swarm optimization algorithm and fuzzy clustering algorithm, the calculation efficiency of the algorithm adopted in this article is obviously improved. The mean shift algorithm is used to divide the color image into several regions. The purpose of this step is to divide the image into related areas according to the color characteristics of the image. Similar to the steps of parallax optimization, the depth map has strict statistical correlation in a segmentation plane, and the plane consistency of the segmentation area of the depth map is used for optimization.

**Figure 4:**
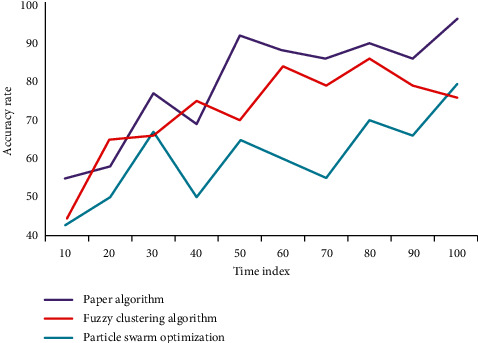
Comparison of accuracy of three algorithms.

**Figure 5:**
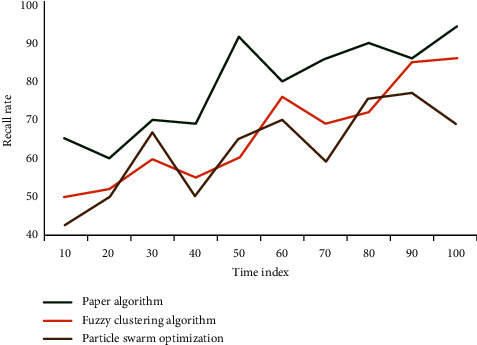
Comparison of recall rates of three algorithms.

Calculate the image's boundary map, then find the path through the image on the boundary map with the least weight, and segment the image at the location with the lowest boundary cost. The image is continuously divided into two parts by searching for the optimal path in both vertical and horizontal directions, resulting in the superpixel of regular grid. The algorithm uses the minimum cut method and the dynamic programming method to find the best path. The former can generate nonregression paths, whereas the latter can generate arbitrary topological paths. When looking for the best path, make sure that no two vertical and horizontal paths cross and that each vertical and horizontal path crosses only once. We use two indicators, accuracy and recall, to verify the effectiveness of this algorithm and compare it to fuzzy clustering and particle swarm optimization algorithms, yielding the following results. The accuracy of the three algorithms is compared in Figure 4. The recall rates of the three algorithms are compared in Figure 5.

According to the proportion and number of segmentation points corresponding to the confidence matching points, it is divided into reliable areas and unreliable areas. For the reliable region, the template parameters are calculated by the weighted least square algorithm according to the confidence matching points. For the unreliable region, the calculated template parameters are unreliable. Using the adjacent good or bad region as the initial template, the final parameters of the unreliable region are obtained through global template parameter optimization. In each iteration, we first rank all the particles according to their contribution to the global optimal solution. In addition, considering the diversity of the population and the fitness value of the optimal solution cannot exceed the average, we will eliminate the particles that are lower than the average fitness value of the whole population.

**Figure 6:**
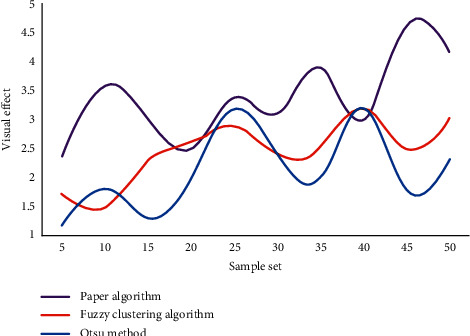
Comparison of image threshold segmentation results.

The depth plane of each image segmentation area passes through the infrared camera, and the infrared light and the sensor receiving the infrared light are in different positions, so the blocked area has no infrared reflection, so the depth value is useless. In addition, the surface of highly bright object does not diffuse the infrared light, which leads to the receiving sensor not receiving the reflected infrared structured light. In order to better test the performance of the algorithm proposed in this article, we compare the proposed method with fuzzy clustering method and Otsu method.  Figure 6 shows the comparison of experimental results of test images.

Different from most image threshold segmentation methods, the method proposed in this article combines the human visual characteristics with the threshold calculation process to automatically extract the target area in the image. The experimental results show that the binary image obtained by this method achieves good visual effect, and its overall visual quality is more in line with the visual perception.

Halftone image is essentially a special kind of binary image. Halftone image pays more attention to layering, whereas general binary image pays more attention to boundary information and contrast. In addition, compared with texture information and multiple gray levels of pixels, general binary images have richer structural information. The extraction method is adaptive and active, without user intervention or any prior information of the image to be segmented. Different input images get different initial contours, and the initial contour contains the target object of the image, which corresponds to the region of visual interest and also accords with the visual cognitive characteristics of human beings. Moreover, the saliency detection algorithm based on frequency domain analysis is simple and easy, with fewer adjustable parameters, and it will not add extra operation time when used in the preprocessing stage before image segmentation. The gray value of each pixel represents its significance, that is, the ability of the pixel to attract the attention of the observer. The larger the significance value, the more likely it is that the pixel is the region of interest, that is, the brighter the region in the saliency map, the potential position of the target object.

**Figure 7:**
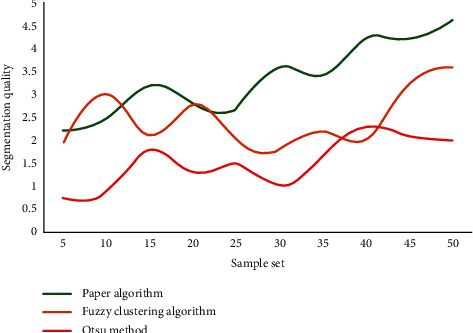
Comparison of segmentation quality of different threshold segmentation methods.

The image pixels are grouped and clustered, and the image plane is divided into a series of “meaningful” areas, based on the similarity criterion of some features or feature sets of images, greatly reducing the amount of data to be processed in later advanced processing stages such as image analysis and recognition, while retaining the information about image structural features. Using the original gray image as a reference image, we compare the visual quality of binary images generated by this method to that of other threshold segmentation methods.  Figure 7 shows the segmentation quality evaluation results of different threshold segmentation methods.

It can be seen from the graph that the method proposed in this article achieves better scores compared with the other two image segmentation methods. Its segmentation quality is higher than that of fuzzy clustering method and Otsu method. This shows that this method has certain advantages compared with other methods, and the binary image generated by this image threshold segmentation method is more in line with human visual perception.

In this section, the active contour model and image segmentation application are updated to include the saliency detection mechanism of human visual cognitive characteristics, and the shape features of the target object in the image can be accurately described using knowledge of the image's bottom features. For various image contents and target objects, different initial contours are constructed adaptively. In the process of image segmentation, two subimages are first constructed in order to reduce the loss of main information from the original image during threshold segmentation, and the calculation of the optimal threshold is transformed into solving an optimization problem. After solving the objective function, the final binary image is created by analyzing the local properties of pixels. Various images were examined and compared with other threshold segmentation methods. Experiments and subjective and objective analysis show that the binary image generated by the proposed image threshold segmentation method is more in line with human visual perception.

## 5. Conclusions

Graphics in modern print advertising design can cross all obstacles, such as language, words, time, space, countries, nationalities, beliefs, and the like, and spread widely all over the world, blending and infiltrating with each other. Graphic vision really realizes the silent spread of advertisements. Modern print advertising plays an increasingly important role in modern political, economic, and social society, and the effect of its advertising communication is closely related to our industry. Only by deeply studying the related elements of print advertising and looking at the development of the whole discipline from the intersection of multiple disciplines, we can have a good grasp of its inherent laws. The visual creation of graphic advertisement is the perfect combination of science and art. Excellent visual creation of graphic advertisement can spread the information of advertisement and can also make the audience get a beautiful visual experience.

This article examines the innovation of graphic design vision in print advertising using image segmentation. From bottom-level image processing to image recognition and understanding, image segmentation is a critical step. Image threshold segmentation is a popular image segmentation method because of its clear physical meaning, high efficiency, and practicality. In the segmentation process, the threshold segmentation method only considers the image's grayscale characteristics and spatial information, ignoring the impact of vision on the segmentation results. In this article, the image's threshold selection process is combined with human vision characteristics, and the original image's information is presented in two subimages. The objective function is optimized by the optimization method to calculate the global optimal threshold of each subimage, and the visual information of each subimage is quantitatively described by human vision characteristics. Finally, the image is subjected to a local adaptive threshold operation based on the image's characteristics, resulting in segmentation results that are more in line with human vision perception. It can handle a wide range of graphics and image segmentation needs.

## Data Availability

The data used to support the findings of this study are included within the article.
